# The Mental Health Bill (2025) for England and Wales: professional and carer consensus statement summarising concerns and unintended consequences from proposed changes to autism and learning disability

**DOI:** 10.1192/bjp.2025.10324

**Published:** 2026-04

**Authors:** Peter Beazley, Regi T. Alexander, John L. Taylor, Bharat Velani, Helen Dewson, Rohit Shankar, Samuel J. Tromans, Mahesh M. Odiyoor, Angela Hassiotis, Ashok Roy, Iain McKinnon, Asif Zia, Andre Strydom, Patrick Keown, Bhathika Perera, Mohsin Khan, Jane McCarthy, Michael Butler, Verity Chester, Lucy Fitton, Kenny Chiu, Andrea Bew, Tadhgh Lane, Tricia Gay, Bob Gay

**Affiliations:** Norwich Medical School, University of East Anglia, Norwich, UK; Hertfordshire Partnership University NHS Foundation Trust, University of Hertfordshire, Hatfield, UK; School of Life & Medical Sciences, University of Hertfordshire, Hatfield, UK; Northumbria University Law School, Faculty of Business & Law, Newcastle upon Tyne, UK; Cumbria, Northumberland, Tyne & Wear NHS Foundation Trust, Newcastle upon Tyne, UK; North London NHS Foundation Trust, London, UK; Norfolk and Suffolk Mental Health Foundation NHS Trust, Norwich, UK; Peninsula School of Medicine, University of Plymouth, Plymouth, UK; SAPPHIRE Group, Department of Population Health Sciences, University of Leicester, Leicester, UK; Leicestershire Partnership NHS Trust, Glenfield, UK; Faculty of Health, Medicine and Society, University of Chester, Chester, UK; Centre for Autism, Neurodevelopmental Disorders and Intellectual Disabilities (CANDDID), Cheshire and Wirral Partnership NHS Foundation Trust, Chester, UK; University College London, London, UK; Coventry and Warwickshire Partnership NHS Trust, Coventry, UK; Population Health Sciences Institute, Academic Psychiatry, Newcastle University, Newcastle upon Tyne, UK; Faculty of Medical Sciences, Newcastle University, Newcastle upon Tyne, UK; Department of Forensic and Neurodevelopmental Science, Institute of Psychiatry, Psychology & Neuroscience, King’s College London, London, UK; West London NHS Trust, London, UK; Sussex Partnership NHS Foundation Trust, Worthing, UK; RADiANT, Hertfordshire Partnership University NHS Foundation Trust, University of Hertfordshire, Hatfield, UK; Carer and Expert by Experience Author, UK

**Keywords:** Mental Health Act, mental health law, autism, learning disability, unintended consequences

## Abstract

The Mental Health Bill, 2025, proposes to remove autism and learning disability from the scope of Section 3 of the Mental Health Act, 1983 (MHA). The present article represents a professional and carer consensus statement that raises concerns and identifies probable unintended consequences if this proposal becomes law. Our concerns relate to the lack of clear mandate for such proposals, conceptual inconsistency when considering other conditions that might give rise to a need for detention and the inconsistency in applying such changes to Part II of the MHA but not Part III. If the proposed changes become law, we anticipate that detentions would instead occur under the less safeguarded Deprivation of Liberty Safeguards framework, and that unmanaged risks will eventuate in behavioural consequences that will lead to more autistic people or those with a learning disability being sent to prison. Additionally, there is a concern that the proposed definitional breadth of autism and learning disability gives rise to a risk that people with other conditions may unintentionally be unable to be detained. We strongly urge the UK Parliament to amend this portion of the Bill prior to it becoming law.

The Mental Health Bill, 2025 (‘the Bill’) was introduced to the House of Lords on 6 November 2024. It proposes to make a number of changes to the Mental Health Act, 1983 (MHA) in England and Wales (separate processes of reform are under way in Scotland,^1^ and a different legislative context exists in Northern Ireland). The process of legislative reform commenced in 2018 with the Independent Review of the Mental Health Act,^2^ which ultimately resulted in the development of the near identical Mental Health Bill in 2022. The previous government never introduced this Bill to parliament.^3^ The aims of the legislative changes are broad, with the most recent impact assessment^4^ identifying 11 separate objectives, many of which relate to the strengthening of patient rights and safeguards.

The present article concerns some of the specific proposed changes relating to autistic people or those with a learning disability^a^ who might require detention and treatment in a hospital. The policy objectives behind these proposals are outlined in the White Paper but, in summary, are to address concerns about autistic people and those with a learning disability being detained for too long or being subject to unnecessary restrictive practices, as well as contemporary concerns about institutional abuse.^5^ The proposed changes would amend Section 3 of the MHA, which governs the process for detentions in hospital for treatment occurring beyond 28 days, such that ‘psychiatric disorder’ (defined as mental disorder other than ‘autism’ or ‘learning disability’) is the only type of mental disorder that can give rise to a need for detention. Detentions could still occur under Section 2 for any type of ‘mental disorder’ (for a maximum of 28 days), and also under Part III of the MHA, which concerns the process of detention in hospital for offenders. Detention under the MHA, regardless of diagnosis, occurs only in relation to risks to self or others.

Several authors and clinicians^6^ have expressed concerns about the potential for unintended consequences because of these proposed changes. This statement has thus been produced as a consensus statement to summarise these concerns. The statement represents the views of a number of academic, legal and clinical professionals, but also includes the voice of a number of carers and experts by experience who share similar concerns, which are particularly highlighted in the expert by experience statement in Appendix 1.

Our concerns are summarised as follows.

## There was no clear mandate for such changes, and their presumed aims are unlikely to be achieved

This key point, highlighted in several articles,^7–9^ is that the proposed changes were not recommended in the Independent Review of the Mental Health Act,^2^ and appeared first in the White Paper.^5^ Qazi et al^10^ have reasonably asked, ‘[w]hy not propose a consultation on change, rather than the removal?’.

It is not clear that changes to primary legislation are the best way to achieve the stated policy objectives. Beazley et al^11^ and Tromans et al^8^ argue that the changes are unlikely to prevent cases of institutional abuse. They highlight that the failings in such institutions have been neither unique to hospital settings nor indeed to autistic people or those with a learning disability, with Tromans et al^8^ concluding that ‘the uncomfortable truth is that poor care can occur in any setting’. Instances of abuse may even increase if the changes result in more people being moved to less well-regulated residential environments.

More generally, because of the wider concerns that will be outlined, we believe that it is unlikely that the proposals will improve parity of esteem for autistic people and those with a learning disability, or reduce stigma.^12^ One of our carer authors remarked that there was a ‘very high risk of further exacerbating the chronic issue of health inequalities experienced by those individuals with a learning disability and autism, resulting in significantly poorer clinical outcomes for these individuals and causing them to unnecessarily experience a much poorer quality of life’.

## The basis for removing autism and learning disability is inconsistent with the conceptualisation of other mental disorders

There is no epidemiological or clinical reason why learning disability and autism should be considered together in the way proposed: they are distinct conditions. Moreover, there are no sound reasons for considering learning disability and autism as sitting apart from the wider legal classification of ‘mental disorder’. As de Villiers^12^ points out, ‘it has never been the case that mental disorder only refers to episodic or psychotic illness’. More generally, Beazley et al^11^ highlight that ‘the practice of drawing a clear line between “mental illness” and “autism” is not necessarily so easy’, and note the complexity in meaningfully attributing specific features of a presentation to a specific condition.

Other papers have identified challenges with the specificity of terms used to differentiate autism and learning disability. For example, two papers^9,11^ challenge the logic that autism and learning disability are ‘untreatable’, highlighting that there are many other examples where the goal of treatment is not to remove a particular condition (for instance, psychological treatment for personality disorder is commonly about increasing the individual’s ability to effectively manage emotional and behavioural symptoms).

## It is inconsistent to remove autism and learning disability from the scope of Part II, but not of Part III

This point has been made by a range of authors,^9,11,13^ who highlight that there is an inherent inconsistency and inequity in allowing a detention to occur under the forensic sections of the Act but not allowing a civil detention under Section 3. The 28-day period allowed under Section 2 is inadequate for meaningful care, treatment or assessment for people who continue to present with serious risks towards the expiry of this section,^8,13,14^ or where ongoing distress clouds the opportunity for assessment of a mental health condition, particularly in the context of the often different presentation of severe mental illness in this population. These were concerns also considered by the Parliamentary Joint Committee.^15^

McKinnon and Keown^14^ have argued that the potential increased use of ‘forensic’ sections under Part III of the Act ‘could have the paradoxical and unintended consequence of detentions under the MHA being more restrictive’ for people with a learning disability. These concerns, as well as the associated concern that the criminal justice system may come under increased pressure to prosecute people with autism and learning disability, were clearly detailed by the joint committee. The potential missed opportunities for treatment and intervention are equally concerning.

One of our carer authors highlighted a concern that, by retaining autism and learning disability within only the ‘forensic’ sections of the Act, there is the risk of unintentionally reinforcing stigmatic beliefs about the dangerousness of autistic people and people with a learning disability.

## It is unclear what will happen to those people who might otherwise have been detained under Section 3

This is a key point, considered in some form by most authors, reflecting concerns around both the potential for unmet care needs and unaddressed risks.

McKinnon and Keown^14^ highlight NHS data indicating that the median length of stay for people with a learning disability is ‘42 days, considerably longer than the 28-day duration of Section 2’. This suggests that there will be a relatively large number of people with a learning disability who will, in practice, be affected by the proposed changes. What happens to those who would currently be detained under Section 3?

Tromans et al^8^ have highlighted the unpreparedness of existing community services to address the care needs and risks presented by people within this group. Velani et al^16^ conducted a survey of 45 English mixed mental health professionals and reported that 76% ‘thought that substantial investment in community services was required in advance of the proposed reforms’. Taylor and Burrell^9,17^ have expressed concern that the proposed processes for supporting the development of community services will probably draw heavily on approaches (such as pooled budgets and joined commissioning) adopted in the ‘failed Transforming Care programme’.

Without recourse to the MHA, it is likely that the Deprivation of Liberty Safeguards (DoLS) framework will be used instead to authorise detentions, at least for the group of people who do not object to their treatment in a hospital. In its present form, the Bill does nothing to prevent the use of DoLS in this way, although the current parliamentary process highlights potential amendments that could also remove this option.^18^

The DoLS framework is, without question, a much more poorly safeguarded option than the MHA.^9,15^ It is also a much more challenging framework under which to manage risk. It offers no access to a second opinion approved doctor (SOAD) to authorise treatment, no ‘nearest relative’ who can initiate discharge, no Article 8 right to an appeal with the corresponding free legal representation and no regular automatic tribunals even if no appeal is made. If the liberty protection safeguards (LPS) are introduced to replace DoLS, such safeguards may be reduced further because authorisation for the LPS moves with the person, rather than needing to be renewed in each new setting. Similar concerns were identified by the joint committee.^15^ Our carer authors expressed a concern that detentions under DoLS might become longer than those under the MHA because of the limited safeguards.

A further disadvantage of an increase in the use of DoLS would be the loss of aftercare provision under Section 117 of the MHA. This provides funding for support in the community following discharge from a Section 3. Our carer authors referred to this as a ‘vital safety net’. Several authors^8,10,11^ have pointed out the implications of removing access to this, with Tromans et al^8^ also highlighting the potential implications for a breach of the Equality Act 2010, particularly if ‘the fabric of social care engagement is not strongly and statutorily designed’. This exclusion might even create a perverse incentive for providers to identify a diagnosis of autism or learning disability to avoid funding obligations under Section 117.

Beyond concerns about DoLS, one of the greatest concerns is the fear that autistic people and those with a learning disability may, instead, be sent to prison because of unmanaged and unnecessary escalation of risk in the community.^8,13,14,19^ We note that research has highlighted the fact that the closure of psychiatric beds, particularly learning disability beds, has been strongly associated with an increase in the prison population.^20^ An increased likelihood of prison sentences was also a concern of our carer authors, who relayed personal experience of this occurring. Our carer authors highlighted this was a concern, not only because of the direct on the individual sent to prison but also because of the stigmatisation associated with criminalisation, both for the individual and also for the wider community of autistic people and those with a learning disability.

The fact that only a relatively small number of regions have a functioning community forensic learning disability team,^21^ a service clearly defined in national service standards,^22^ raises a particular concern that any associated hospital closures that follow the proposed changes would mean that some areas may be left with no functioning services with relevant professional expertise in risk management of learning disability, thus increasing the likelihood of risks escalating without effective intervention and support. There are particular concerns about people awaiting trial in the community who might otherwise be managed via detention under Section 3.^14^

It is important to emphasise the potential impact on public protection based on unmanaged risks. Of course, most autistic people or those with a learning disability present no wider risk to others. However, in some cases there are risks including fire-setting, sexual violence and stalking. Some ‘special interests’ in autism can also cause concern (e.g. poisons or explosives). These behaviours occur for a complex range of reasons, and effective risk management is important to assure public safety, including that of carers. When such risks begin to escalate, a detention under Section 3 is a key mechanism to prevent further development with more serious consequences.

Beazley et al^11^ highlighted a particularly concerning scenario for people who commit a serious offence but who are unable to be prosecuted for it for any reason. A ‘hospital order’ under Section 37 of the MHA generally^b^ relies on a successful prosecution occurring. Plenty of prosecutions are not pursued for evidential reasons, or because the Crown Prosecution Service judges them not to be in the public interest. For people with profound impairments, their probable unfitness to plead or wider difficulties in engaging in trial proceedings may also be relevant and, even if such proceedings are brought, the underlying cognitive impairment may cause a failure in the underlying *mens rea*, meaning that charges are dropped. This could leave a person with no prison sentence, no access to the MHA and, if the person objects, no access to DoLS. If the underlying behaviour means that the person also loses their residence (this might occur if the initial incident is, for example, an assault on a staff member in a residential home), it could leave a group of people with literally nowhere to go.

Finally, if increased numbers of autistic people or those with a learning disability move into community residential or care settings, it will be vital to improve the capacity, governance and quality of housing and care provision at these locations, as well as associated community NHS services. The joint committee indicated they had ‘serious concerns that the deficit in community care provision has the potential to derail these reforms and lead to worse outcomes for this group’.^15^ One key factor is that, while care homes will typically be registered with the Care Quality Commission, many supported living environments are not. One of our carer authors, reflecting on their son’s experience, noted that ‘the level of squalor and misery that was deemed acceptable is unbelievable when I look back on it now’. Echoing this, a pilot study that reviewed stakeholder views concerning supported living and residential care settings highlighted a wide range in the quality of care provided.^23^

## The definition of ‘autism’ is too broad (and so the definition of ‘psychiatric disorder’ is consequently too narrow)

Beazley^24^ has highlighted a specific concern about the breadth of the proposed definition of ‘legal autism’ in the Bill, noting that this is much broader in scope than any clinical conceptualisation. This paper raises the concern that, because ‘psychiatric disorder’ is ‘defined primarily by what it is not (i.e. legal autism or learning disability)’, there is a risk that conditions other than autism (including, but by no means limited to, personality disorder) could be argued as meeting the definition of ‘legal autism’ and thus be excluded from the scope of a Section 3 detention. The paper raises a particular concern about the resultant necessity for tribunals to have an increased focus on ‘mental disorder’ (as opposed simply to ‘nature’ or ‘degree’), particularly given the fact that presenting an autism diagnosis that subsumes or overlaps with ‘nature’ might become a compelling line of argument for advocates arguing for discharge. This is a particular concern given that autistic people are known to have high rates of co-occurring psychiatric conditions.^25^ The briefing by the Parliamentary Office of Science and Technology^26^ acknowledges that ‘[m]ental health disorders can present differently in autistic people, and combined with communication difficulties this can make disorders harder to diagnose’. In a situation with comorbid or overlapping features, who determines where the boundaries of the excluded ‘legal autism’ and remaining ‘psychiatric disorder’ begin and end?

Wong^27^ has argued for the need for ‘definitional width’ more generally from a legal standpoint, concluding that ‘a wide definition allows for requisitely flexible approaches to treatment administration under practical complexities’. Certainly, creating legal definitions of clinical problems, disorders or conditions is an inherently complex process, with lessons to be drawn from the ‘Dangerous and Severe Personality Disorder’ concept introduced following the last set of MHA reform.^11^ If legislators wish to avoid the unintended consequences arising from adopting a broad clinical definition for a legal purpose (particularly one with an exclusionary function), it is important that development of the corresponding definitions and conceptualisations is underpinned by additional research.

### Implications for future legislative reform

The summary of concerns we have identified speaks to key issues of fairness and justice. We note that our concerns mirror many of those identified by Tromans et al,^6^ who considered the views in a sample of 82 psychiatrists. In this sample, over half reported disagreement with the proposed changes, with greater concerns being noted by more senior psychiatrists and those working in in-patient settings.

It is also important to note that other jurisdictions that have implemented such changes have at least partially rolled them back. Several authors^6–9,11,17,27,28^ highlighted the experience in New Zealand, which is the only common-law jurisdiction to have implemented such changes. Taylor and Burrell^7,9,17^ describe the resultant ‘legislative gap’, where people with a learning disability were left with no effective community care and, as a result, an increased number went on to commit serious offences, resulting in an increased number being sent to prison. Subsequent legislative changes to address these issues have resulted in ‘net widening, with more rather than fewer people with intellectual disabilities becoming subject to compulsory care in detention’.

The authors of this statement are united in their desire to see improvements in the care and treatment of autistic people and those with a learning disability. We recognise that the use of detention under Section 3 is a significant intervention with a person’s civil liberties and rights, but this is no less the case for an autistic person or somebody with a learning disability as it is for somebody with another condition that gives rise to a need for care and treatment in a restricted setting. All of us would prefer to live in a world where detention under the MHA was unnecessary entirely, but this desire does not reflect the nature of reality where such detentions can be often viewed as the ‘least worst’ of a range of pragmatic solutions to manage high levels of risks to self and/or others.

What are the answers or alternatives? While the answers to this question lie beyond the scope of this paper, we collectively agree that such detentions are likely to become less necessary if increased resourcing is allocated to appropriate community support for autistic people and those with a learning disability, and that such support is characterised by a qualified and competent workforce, suitable supported accommodation and meaningful occupational activities. However, better community care will not entirely remove the need for assessment and treatment in hospital of a relatively small proportion of autistic people and those with a learning disability who present with ambiguous or unclear clinical presentations, or significant risks, and where proper assessment and treatment will take longer than 28 days. Presently, therefore, we advocate keeping autism and learning disability within the scope of Section 3, because of the likely unintended consequences and adverse impact on people affecting their quality of life, liberty and access to treatment and care.

Box 1 Key points

- The Mental Health Bill (2025), as drafted, will remove autistic people and those with a learning disability from the scope of Section 3 of the Mental Health Act, 1983. This will limit civil detentions to 28 days, under Section 2 only.
- A number of authors have expressed concerns about these changes. A range of potential unintended consequences have been identified. This paper provides a consensus statement from professionals and carers who are concerned about such proposals.
- It seems unlikely that the changes will result in their anticipated aims: autistic people and those with a learning disability may instead be more likely to be sent to prison more frequently and be more likely to be detained under regimes affording fewer safeguards.
- Alternatively, autistic people or those with a learning disability who present with significant risks and are supported in the community may be more likely to act on these risks, leading to increased police involvement and risks to the public.
- People who do not have autism or a learning disability may also be impacted, because the proposed legal definitions of ‘autism’ and ‘learning disability’ are so broad.
- Significant legal complications may arise where there is a need to detain someone who has another condition that overlaps or intersects with their autism or learning disability. This is expected to occur relatively frequently.

## Data Availability

Data availability is not applicable to this article because no new data were created or analysed in this study.

## Footnotes

a. Although several authors use the term ‘intellectual disability’, the present article uses ‘learning disability’ to reflect the terminology of the Bill.

b. The word ‘generally’ is intentional. There is an alternative process via s.5 of the Criminal Procedure Insanity Act (1964) that could result in a hospital order disposal for a person who is not fit to plead or who is found ‘not guilty by reason of insanity’. However, this procedure is presently very infrequently used.

## Relevance statement

The proposed changes to the Mental Health Act concerning autism and learning disability will result in a range of unintended consequences having relevance for all psychiatrists working with patients subject to civil detention. People with autism and learning disability may be more likely to be sent to prison, or detained under less well-safeguarded regimes. More broadly, the changes may make it harder to justify detention, particularly for people with conditions that co-occur with autism and learning disability, or where similar symptoms are demonstrated.

## Appendix 1: expert by experience perspective

As a parent of a learning-disabled adult with autism and mental health issues, I have had some considerable experience in this area. I have seen my son in both a mental hospital and in ‘care in the community’.

It is a common assumption, and one that I held myself before my son spent time in hospital, that any living situation is preferable to hospital and that hospital is not just a deprivation of freedom, but a last resort, end of hope option when everything else fails. Instead, and in my son’s case, it has been a temporary place of safety and genuine care, which has enabled him to recover mentally to a level whereby he is now able to continue his life back in the community.

What I feel is not often appreciated is that the enormous levels of fear and anxiety felt by some autistic, learning-disabled people, through trying to live in the community, can be so overwhelming that their behaviours escalate and they tip over into mental illness, and sometimes offending behaviour. Having watched this happen to my son, I can absolutely attest that this amounts to no quality of life whatsoever.

Community placements are great when they work but, in my experience, they are often woefully inadequate. My son’s last placement was abusive and unsafe. He was dirty, undernourished, angry, sad, confused and desperate. It is in this environment that the autistic, learning-disabled person is asked to make sense of a staggeringly complex world. This makes them not only desperate in themselves but very, very vulnerable. I bless the day that my son was rescued from that cruel living environment (via the criminal justice system) and transferred to a medium-secure mental health clinic. Through proper mental health assessments, and insightful person-centred care, the hospital has brought him back to the person he was.

I am very aware that the fear is that, in some cases, autistic or learning-disabled people can be in hospital for too long, as in ’shut away and forgotten’, but I feel that this is a separate issue and one that should not mean that they have no access at all to the help they sometimes need in a hospital setting. These people shouldn’t have to descend so far that they become involved with the criminal justice system before they get the help that they need.
